# Colorectal cancer cells suppress CD4+ T cells immunity through canonical Wnt signaling

**DOI:** 10.18632/oncotarget.14834

**Published:** 2017-01-27

**Authors:** Xuan Sun, Suoning Liu, Daguang Wang, Yang Zhang, Wei Li, Yuchen Guo, Hua Zhang, Jian Suo

**Affiliations:** ^1^ Department of Gastrointestinal Surgery, First Hospital of Jilin University, Changchun, Jilin province, China

**Keywords:** canonical Wnt signaling, negatively regulate, CD4+T cells immunity, colorectal cancer

## Abstract

Understanding how colorectal cancer escapes from immunosurveillance and immune attack is important for developing novel immunotherapies for colorectal cancer. In this study we evaluated the role of canonical Wnt signaling in the regulation of T cell function in a mouse colorectal cancer model. We found that colorectal cancer cells expressed abundant Wnt ligands, and intratumoral T cells expressed various Frizzled proteins. Meanwhile, both active β-catenin and total β-catenin were elevated in intratumoral T cells. *In vitro* study indicated that colorectal cancer cells suppressed IFN-γ expression and increased IL-17a expression in activated CD4^+^ T cells. However, the cytotoxic activity of CD8^+^ T cells was not altered by colorectal cancer cells. To further evaluate the importance of Wnt signaling for CD4^+^ T cell-mediated cancer immunity, β-catenin expression was enforced in CD4^+^ T cells using lentiviral transduction. In an adoptive transfer model, enforced expression of β-catenin in intratumoral CD4^+^ T cells increased IL-17a expression, enhanced proliferation and inhibited apoptosis of colorectal cancer cells. Taken together, our study disclosed a new mechanism by which colorectal cancer impairs T cell immunity.

## INTRODUCTION

Colorectal cancer (CRC) is a major worldwide health problem owing to its high prevalence and mortality rates [1]. Immune surveillance and responses have been shown to prevent and inhibit the initiation and progression of CRC [2]. However, Immune escape is still an intriguing problem involved in CRC pathogenesis and metastasis [3]. Thorough understanding of anti-CRC immunity will shed new lights on developing effective therapeutics for CRC.

Infiltration of immune cells into tumors has been associated with destruction of tumor cells, reduction of tumor burden, and better clinical prognosis [4]. Among various immune cells, T cells are especially important for suppressing and eliminating CRC cells. Tumor antigen-specific T cells can be detected in the blood and bone marrow of 30% to 40% of patients with CRC [5, 6]. CRC patients with more T cell infiltration have better outcomes [4, 7, 8]. In particular, TNF-α and IFN-γ are both critical for T cells to protect CRC patients through inhibition of cancer cell proliferation and induction of apoptosis [9–17]. However, how T cell function is regulated in CRC sites has not been completely understood.

In this study, we examined the effect of CRC cell-derived Wnt proteins on intratumoral T cell function in a murine CRC implantation model. We found that CRC cell lines expressed an array of Wnt proteins, while intratumoral T cells increased various Frizzled (FZD) proteins as Wnt receptors. Meanwhile, both active β-catenin and total β-catenin were elevated in intratumoral T cells in comparison with splenic T cells. We further found that CRC cell-derived Wnt proteins suppressed IFN-γ expression and increased IL-17a expression in activated CD4^+^ T cells. However, the cytotoxic activity of CD8^+^ T cells was not altered by CRC cells. Enforced expression of β-catenin in intratumoral CD4^+^ T cells increased IL-17a expression. Moreover, enforced expression of β-catenin in intratumoral CD4^+^ T cells enhanced proliferation and inhibited apoptosis of CRC cells. Taken together, our study disclosed a new mechanism by which CRC impairs T cell immunity.

## RESULTS

### CRC cell lines express various Wnt proteins

**Figure 1:**
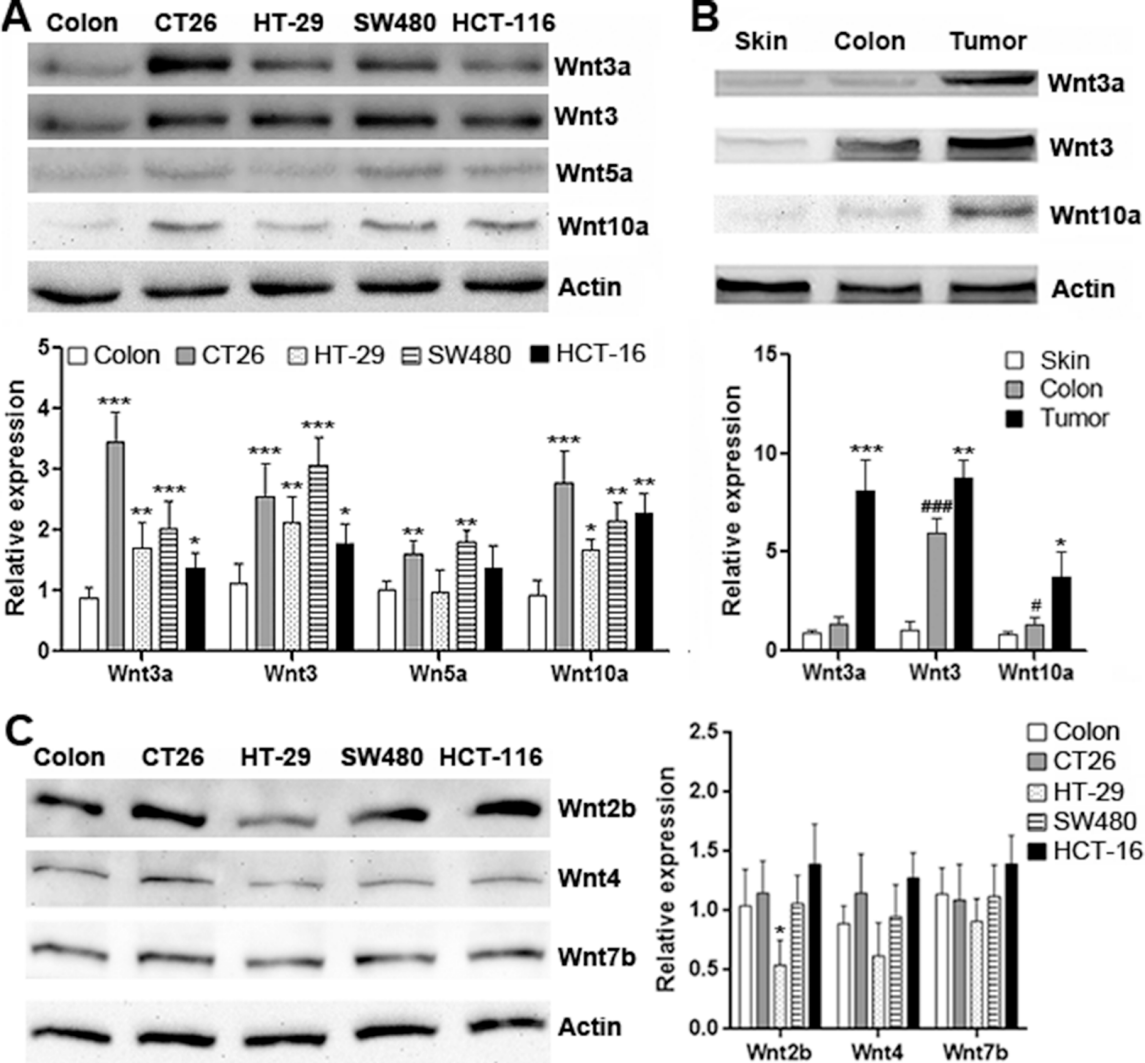
Expression of Wnt proteins in CRC cell lines (**A**) Expression of Wnt3a, Wnt3, Wnt5a and Wnt10a in mouse and human CRC cell lines. Upper panel: representative Western blot images. Lower panel: statistical analysis for expression of each Wnt protein. *N* = 6 per group. *p < 0.05; **p < 0.01; ***p < 0.001 compared with normal colon tissue. (**B**) Expression of Wnt3a, Wnt3 and Wnt10a in normal tissues and CT26.CL25 tumor grafts. Upper panel: representative Western blot images. Lower panel: Statistical analysis for expression of each Wnt protein. Skin: normal skin tissue. Colon: normal colon tissue. Tumor: tumor grafts. *N* = 4 per group. *p < 0.05; **p < 0.01; ***p < 0.001 compared with normal colon tissue. ^###^*p* < 0.05; ^###^*p* < 0.001 compared with normal skin tissue. (**C**) Expression of Wnt2b, Wnt4 and Wnt7b. Left panel: representative Western blot images. Right panel: Statistical analysis for expression of each Wnt protein. *N* = 3 per group. *p < 0.05 compared with normal colon tissue.

Elevated expression of Wnt3, Wnt3a and Wnt10a in CRC has been disclosed by previous studies [18–20]. We firstly identify the expression of Wnt3, Wnt3a, Wnt5a and Wnt10a in several CRC cell lines. As shown in Figure 1A, in comparison with normal mouse colon tissue, expression of Wnt3, Wnt3a and Wnt10a was significantly increased in CT26.CL25, HT-29, SW480 and HCT-116 cells, whereas Wnt5a expression was only moderately increased in CT26.CL25 and SW480 cells. Among these cell lines, CT26.CL25 expressed high levels of Wnt3, Wnt3a and Wnt10a. Therefore, CT26.CL25 cells were utilized in the following experiments. To determine the *in vivo* expression of Wnt proteins in CT26.CL25 cells, CT26.CL25 cells were subcutaneously inoculated into Rag1^−/−^ mice to form tumor grafts. Western blot analysis showed that Wnt3, Wnt3a and Wnt10a were indeed expressed in the tumor grafts, and their expression levels were higher than those in normal subcutaneous tissue and normal mouse colon (Figure 1B). To determine the expression of Wnts in non-CRC cell types in the tumor grafts, we sorted host-derived endothelial cells, fibroblasts, macrophages and dendritic cells and tested mRNA levels of Wnt3, Wnt3a, Wnt5a and Wnt10a in these cells. In comparison with implanted CT26.CL25 cells, non-CRC cell types expressed very low or no Wnt3, Wnt3a, Wnt5a and Wnt10a, suggesting that implanted CRC cells were the major source of these Wnts (Supplementary Figure 1). In addition, other Wnts that were reported to be present in both normal colon tissue and CRC, such as Wnt2b, Wnt4 and Wnt7b [21], were all expressed in these CRC cell lines, although at different levels (Figure 1C).

### Intratumoral T cells express FZD proteins

**Figure 2:**
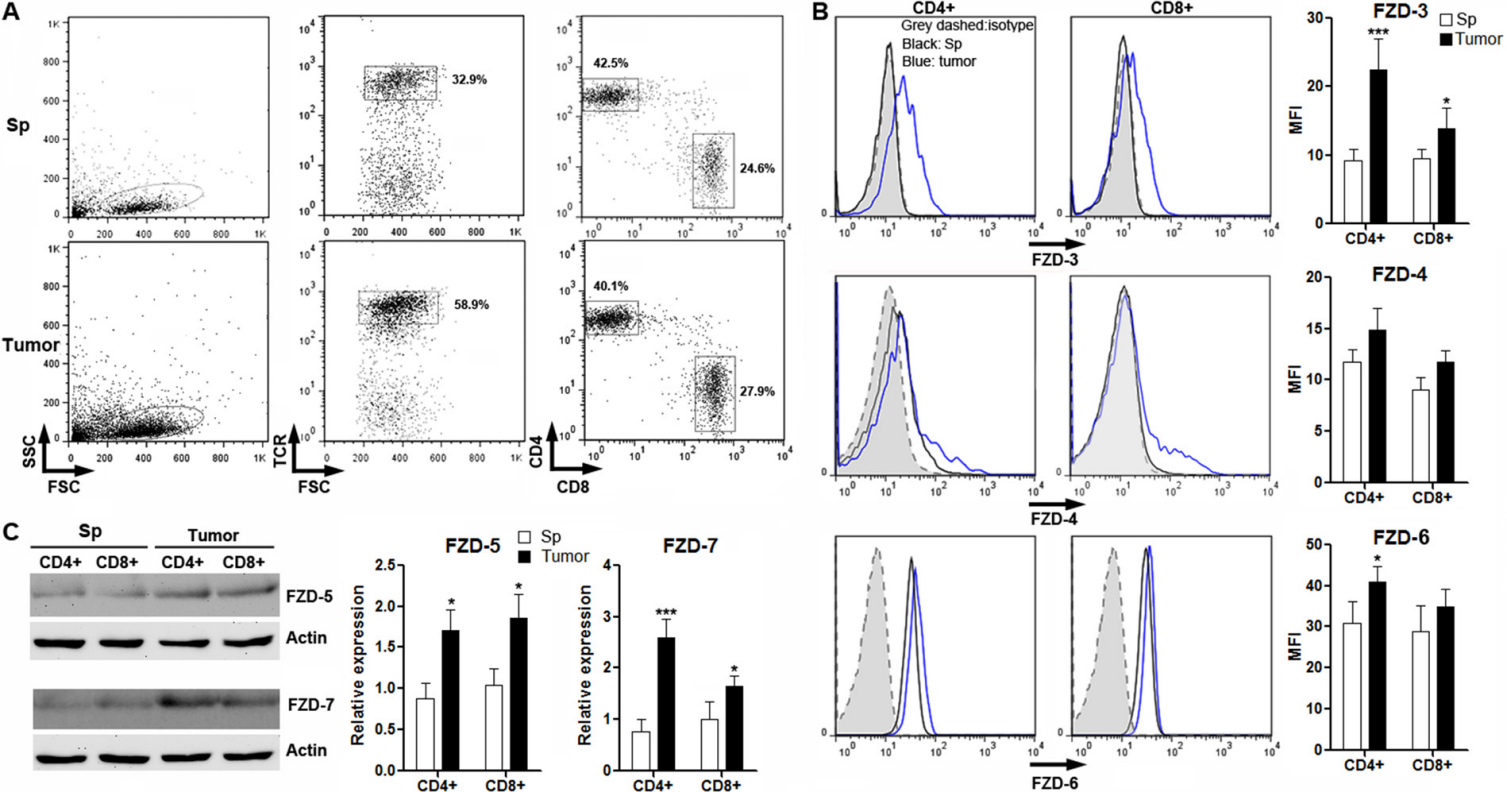
Expression of FZD protein in intratumoral T cells (**A**) Gating strategy for splenic and intratumoral CD4^+^ and CD8^+^ T cells. Numbers in the plots are the percentages of gated populations. Sp: spleen. Tumor: tumor grafts. This is a representative of three independent experiments. (**B**) Expression of FZD-3, FZD-4 and FZD-6 on T cells were determined by flow cytometry. Left panel: representative histograms. Right panel: statistical analysis for mean fluorescence intensity of each FZD protein. **(C**) Expression of FZD-5 and FZD-7 in T cells were determined by Western blot. Left panel: representative image. Right panel: statistical analysis for each FZD protein. Sp: spleen. Tumor: tumor grafts. *N* = 3 per group. *p < 0.05; ***p < 0.001 compared with splenic T cell subsets.

A previous research has outlined expression of FZD proteins in resting and effector T cells *in vitro* [22]. So we also checked expression of these FZD proteins in intratumoral T cells to characterize the potential Wnt signaling in anti-tumor T cells. To do so, splenic CD3^+^ T cells were enriched by flow cytometry from BALB/C mice and were transferred into tumor-bearing Rag1^−/−^mice. Three weeks after transfer, T cells were localized in proximity to CRC cells in tumor grafts (Supplementary Figure 2). FZD proteins were determined in T cells isolated from spleens and tumor grafts. As shown in Figure 2A, TCR^+^CD4^+^ and CD8^+^ donor-derived T cells were present in both spleens and tumor grafts. Flow cytometry and Western blot analysis indicated that splenic T cells expressed very low levels of these FZD proteins except for FZD-6. However, FZD-3, FZD-5 and FZD-7 were increased in intratumoral CD4^+^ and CD8^+^ T cells in comparison with splenic counterparts, whereas FZD-6 was only slightly increased in intratumoral CD4^+^ T cells. FZD-4 was just increased in a small subpopulation of either CD4^+^ or CD8^+^ T cells (Figure 2B and 2C). Taken together, our data suggested that intratumoral T cells have higher expression of FZD proteins than splenic T cells.

### β-catenin is activated in intratumoral T cells

**Figure 3:**
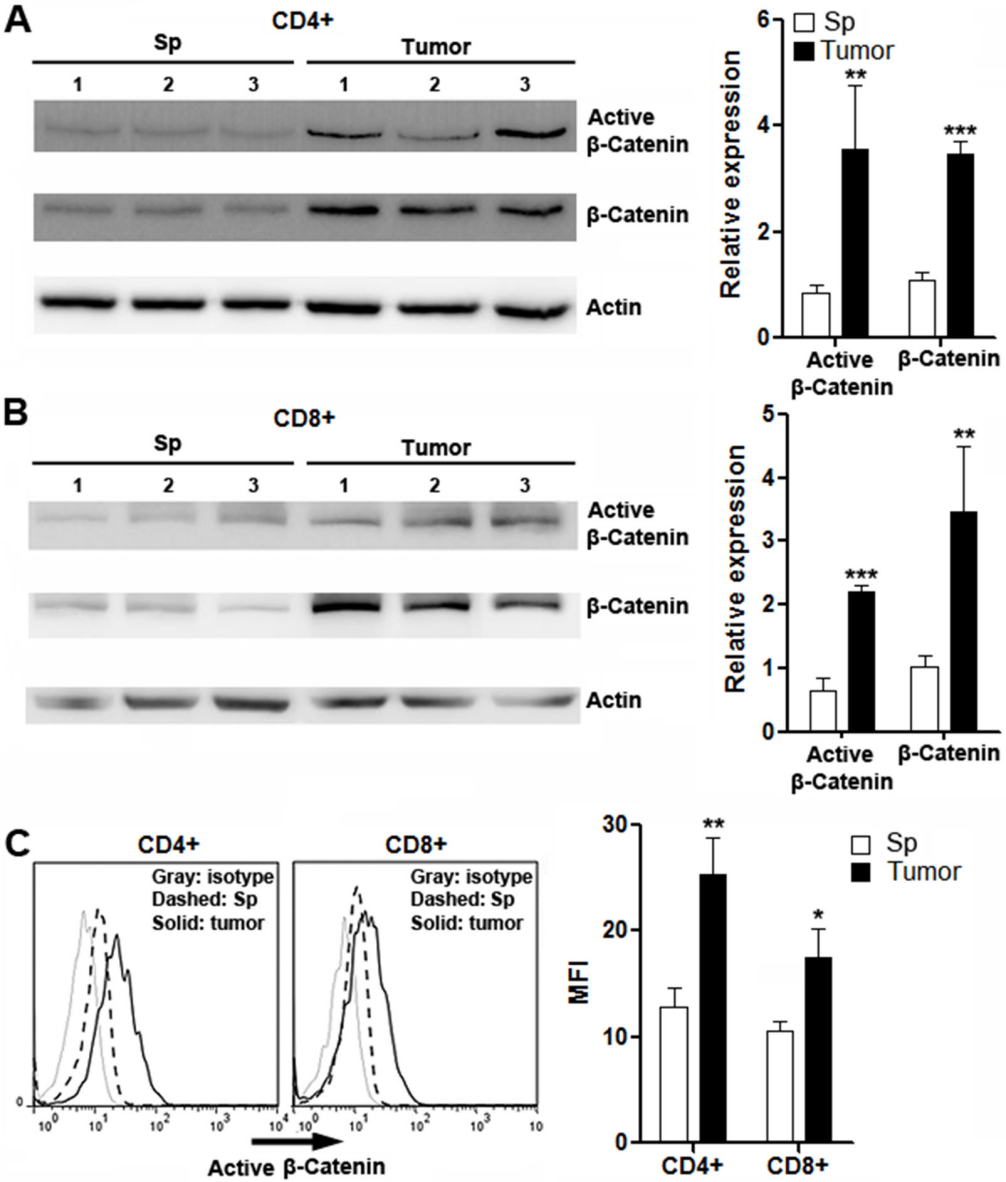
Expression of active β-catenin and total β-catenin in intratumoral T cells (**A** and **B**) Expression of active β-catenin and total β-catenin in splenic and intratumoral CD4^+^ (A) and CD8^+^ (B) T cells were determined by Western blot. Left panels: representative Western blot images. Right panels: statistical analysis. (**C**) Expression of active β-catenin in splenic and intratumoral CD4^+^ and CD8^+^ T cells were determined by flow cytometry. Left panel: representative histograms. Right panel: statistical analysis for mean fluorescence intensity of active β-catenin. Sp: spleen. Tumor: tumor grafts. *N* = 3 per group. *p < 0.05; **p < 0.01; ***p < 0.001 compared with splenic T cell counterparts.

To further define the activation status of canonical Wnt signaling in T cells, both active β-catenin and total β-catenin were detected in splenic and intratumoral T cells. We found that intratumoral CD4^+^ and CD8^+^ T cells expressed remarkably higher active β-catenin and total β-catenin than splenic T cell subsets (Figure 3A and 3B), suggesting β-catenin activation in intratumoral T cells. It was also noted that β-catenin expression was very weak in splenic T cells, which was consistent with previous reports [23, 24]. The increase of active β-catenin in intratumoral T cells was also confirmed by flow cytometry analysis (Figure 3C).

### CT26.CL25 cells induce activation of β-catenin in activated T cells

**Figure 4:**
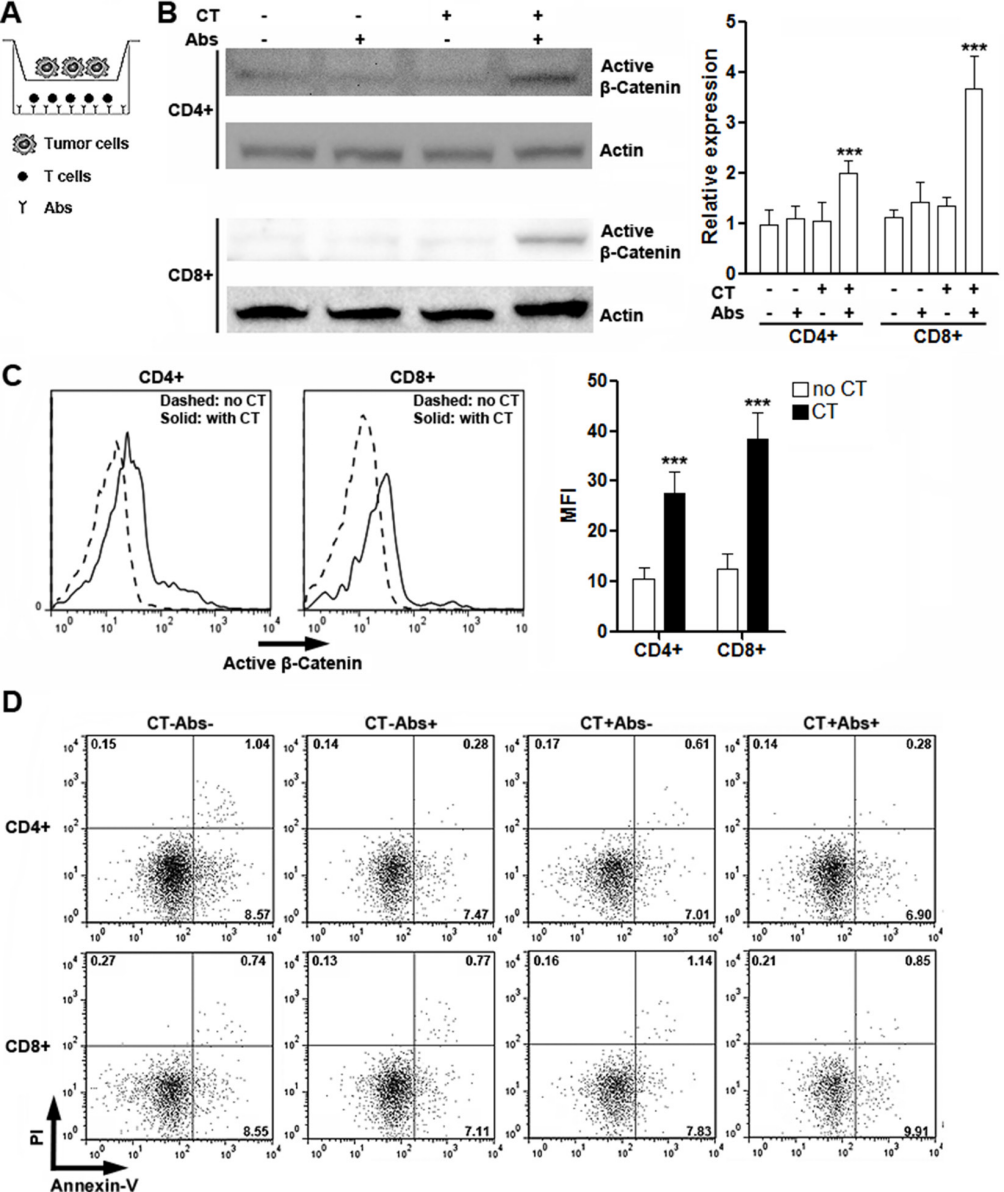
CT26.CL25 cells induce activation of β-catenin in activated T cells (**A**) Illustration of co-culture system. (**B**) Expression of active β-catenin in cultured splenic CD4^+^ and CD8^+^ T cells was determined by Western blot. Left panels: representative Western blot images. Right panels: statistical analysis for active β-catenin. CT: CT26.CL25 cells. Abs: agonistic antibodies. *N* = 6 per group. ***p < 0.001 compared with T cell culture without CT26.CL25 cells and agonistic antibodies. (**C**) Expression of active β-catenin in activated splenic CD4^+^ and CD8^+^ T cells was determined by flow cytometry. Note that T cells shown here are all stimulated with agonistic antibodies. Left panel: representative histograms. Right panel: statistical analysis. No CT: without CT26.CL25 cells. CT: co-culture with CT26.CL25 cells. *N* = 6 per group. ***p < 0.001 compared with “No CT” group. (**D**) Flow cytometry analysis of T cell apoptosis in culture. Numbers in the quadrants are percentages of corresponding gated populations. This is a representative of two independent experiments.

To identify the role of CRC cells in inducing β-catenin activation in T cells, we co-cultured CT26.CL25 cells with T cells in the presence or absence of agonistic antibodies in Transwell plates (Figure 4A), followed by detection of active β-catenin in T cells. As shown in Figure 4B, either agonistic antibodies or tumor cells alone was unable to induce up-regulation of active β-catenin in T cells, while co-existence of agonistic antibodies and tumor cells increased expression of active β-catenin, suggesting that both T cell activation and tumor cell-derived soluble factors are indispensable for efficient Wnt signaling in T cells. The up-regulation of active β-catenin was further confirmed by flow cytometry analysis (Figure 4C). Moreover, co-culture with tumor cells had no remarkable impact on T cell apoptosis (Figure 4D), suggesting that potential changes of T cell functions might not be resulted from alteration of T cell viability.

### CT26.CL25 cells bias CD4^+^ T cell polarization towards Th17 via Wnt signaling

**Figure 5:**
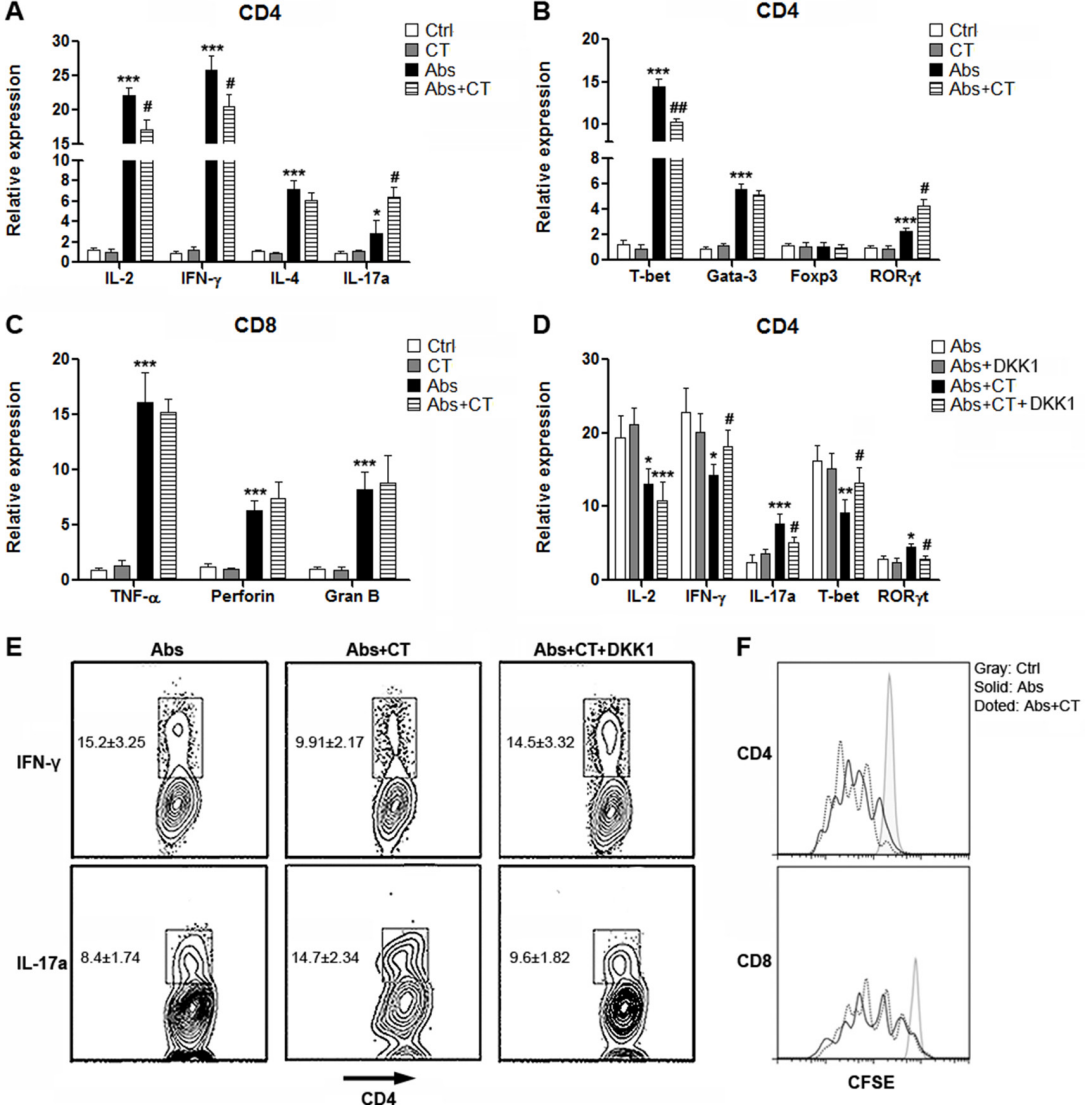
CT26.CL25 cells bias CD4^+^ T cell polarization towards Th17 via Wnt signaling (**A** and **B**) Expression of cytokines (A) and master regulators (B) in cultured CD4^+^ T cells were determined by real-time PCR. Ctrl: untreated T cells. CT: T cells co-cultured with CT26.CL25 cells. Abs: agonistic antibody-stimulated T cells. Abs+CT: agonistic antibody-stimulated T cells co-cultured with CT26.CL25 cells. (**C**) Expression of cytotoxic mediators in cultured CD8^+^ T cells were determined by real-time PCR. (**D**) Expression of cytokines and master regulators in cultured CD4^+^ T cells were determined by real-time PCR. Abs: agonistic antibody-stimulated T cells. Abs+DKK1: agonistic antibody-stimulated T cells in the presence of DKK1. Abs+CT: agonistic antibody-stimulated T cells co-cultured with CT26.CL25 cells. Abs+CT+DKK1: agonistic antibody-stimulated T cells co-cultured with CT26.CL25 cells and DKK1. (**E)** Expression of IFN-γ and IL-17a were measured by flow cytometry analysis. Numbers in the plots are percentages of gated populations. (**F**) CFSE dilution in proliferative CD4^+^ T cells. *N* = 6 per group. For A to C, *p < 0.05; **p < 0.01; ***p < 0.001 compared with Ctrl group. #*p* < 0.05; ##*p* < 0.01 compared with Abs group. For D, *p < 0.05; **p < 0.01; ***p < 0.001 compared with Abs group. #*p* < 0.05 compared with Abs+CT group.

To determine the influences of CRC cells on T cell immunity, we co-cultured CT26.CL25 cells with T cells in the presence or absence of agonistic antibodies in Transwell plates before detection of cytokine production and master regulator expression. For CD4^+^ T cells without agonistic antibody stimulation, co-culture with CT26.CL25 cells could not their change cytokine production and master regulator expression, suggesting that CT26.CL25 cells could not induce activation of resting CD4^+^ T cells (Figure 5A and 5B). In the presence of agonistic antibodies, CT26.CL25 cells inhibited expression of IL-2 and IFN-γ but increased IL-17a production in CD4^+^ T cells (Figure 5A). Consistently, in the presence of agonistic antibodies, CT26.CL25 cells lower the expression of T-bet while up-regulating RORγt (Figure 5B). Expression of IL-4 or Gata-3 was not altered (Figure 5A and 5B). For CD8^+^ T cells, no significant changes were observed in the presence of CT26.CL25 cells (Figure 5C). Our findings suggested that CT26.CL25 cells might predominantly influence CD4^+^ T cell function. So in the following experiments we focused on CD4^+^ T cells only.

To examine the role of Wnt signaling in CRC cell-induced alterations of CD4^+^ T cells, we added Wnt antagonist DKK1 into the co-culture system. As shown in Figure 5D, in the absence of CT26.CL25 cells, DKK1 itself did not change the expression of cytokines or master regulators, suggesting that Wnt signaling did not function during CD4^+^ T cell activation. However, DKK1 abolished the effects of CT26.CL25 cells on CD4^+^ T cell activation, demonstrated by restored expression of IFN-γ and T-bet, as well as down-regulation of IL-17a and RORγt. The changes of IFN-γ and IL-17a were also confirmed by flow cytometry analysis (Figure 5E). Furthermore, CT26.CL25 cells did not alter the proliferation of activated CD4^+^ T cells (Figure 5F).

### Enforced expression of β-catenin in CD4^+^ T cells through lentiviral transduction

**Figure 6:**
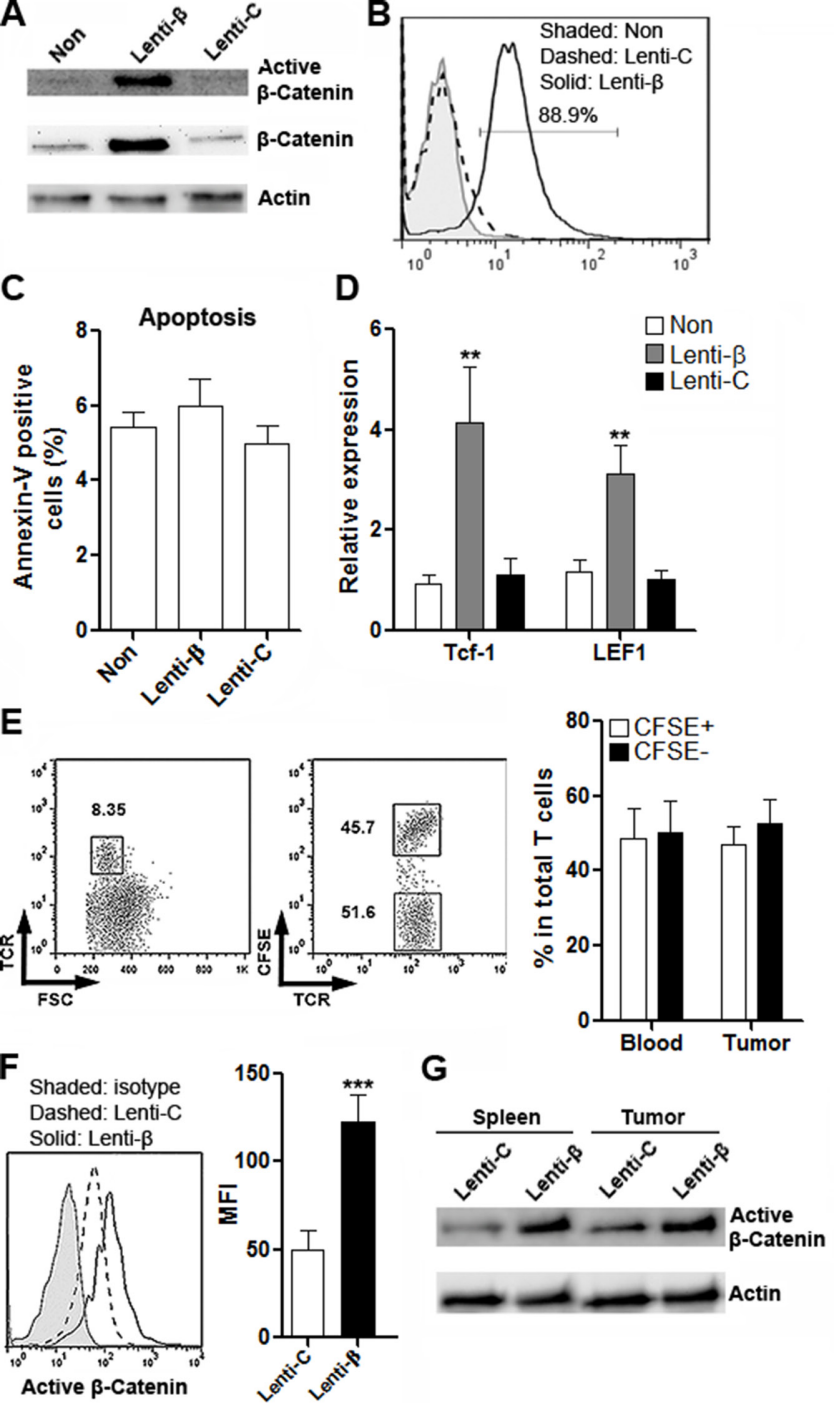
Enforced expression of β-catenin in CD4^+^ T cells through lentiviral transduction (**A**) Expression of active β-catenin and total β-catenin in CD4^+^ T cells after lentiviral transduction was determined by Western blot. This is a representative of two independent experiments. Non: non-transduced T cells. Lenti-β: T cells transduced with β-catenin Lentiviral Activation Particles. Lenti-C: T cells transduced with control lentiviral particles. (**B**) Expression of active β-catenin in T cells was detected by flow cytometry. This is a representative of two independent experiments. (**C**) T cells apoptosis after lentiviral transduction. *N* = 4 per group. (**D**) Expression of Tcf-1 and LEF1 in T cells were evaluated by real-time PCR after lentiviral transduction. *N* = 6 per group. **p < 0.01 compared with “Non” group. (**E**) Proportions of CFSE^-^ and CFSE^+^ T cells in tumor grafts were evaluated by flow cytometry. Left panel: representative dot plots. Right panel: statistics. Numbers in the plots are proportions of corresponding gated populations. *N* = 3 per group. (**F**) Expression of active β-catenin in donor-derived intratumoral T cells was determined by flow cytometry. Left panel: representative histograms. Right panel: statistics of MFI. *N* = 3 per group. ***p < 0.001. (**G**) Expression of active β-catenin in donor-derived splenic and intratumoral T cells was determined by Western blot. This is a representative of two independent experiments.

To clarify the role of Wnt signaling in T cell immunity *in vivo*, expression of β-catenin was enforced in CD4^+^ T cells. These CD4^+^ T cells contained CRC-reactive T cells, because they were sorted from the draining lymph nodes of BALB/C mice which were subcutaneously inoculated with CT26.CL25 cells. Transduction of β-catenin Lentiviral Activation Particles (Thereinafter Lenti-β) prompted robust expression of β-catenin as well as active β-catenin (Figure 6A and 6B). T cell apoptosis was not influenced by lentirivral transduction or β-catenin up-regulation (Figure 6C). Expression of two Wnt signaling target genes, Tcf-1 and LEF-1, was notably increased in Lenti-β-transduced T cells, further confirming the activation of canonical Wnt signaling (Figure 6D). To measure the transmigration of T cells into the colon cancer site, T cells transduced with control lentivirus (Thereinafter Lenti-C) were labeled with CFSE before adoptive transfer with T cells transduced with Lenti-β into tumor-bearing Rag1^−/−^ mice. One week after transfer, the proportions of CFSE^+^ and CFSE^−^ T cells were detected in intratumoral T cells (Figure 6E). As shown in Figure 6E, almost equal percentages of CFSE^+^ and CFSE^−^ T cells were found in intratumoral T cells, suggesting that β-catenin expression did not interfere with T cell migration. Furthermore, active β-catenin expression level was markedly higher in Lenti-β-transduced T cells in both spleens and tumor grafts (Figure 6F to Figure 6G).

### Wnt signaling in CD4^+^ T cells favors CRC graft growth

**Figure 7:**
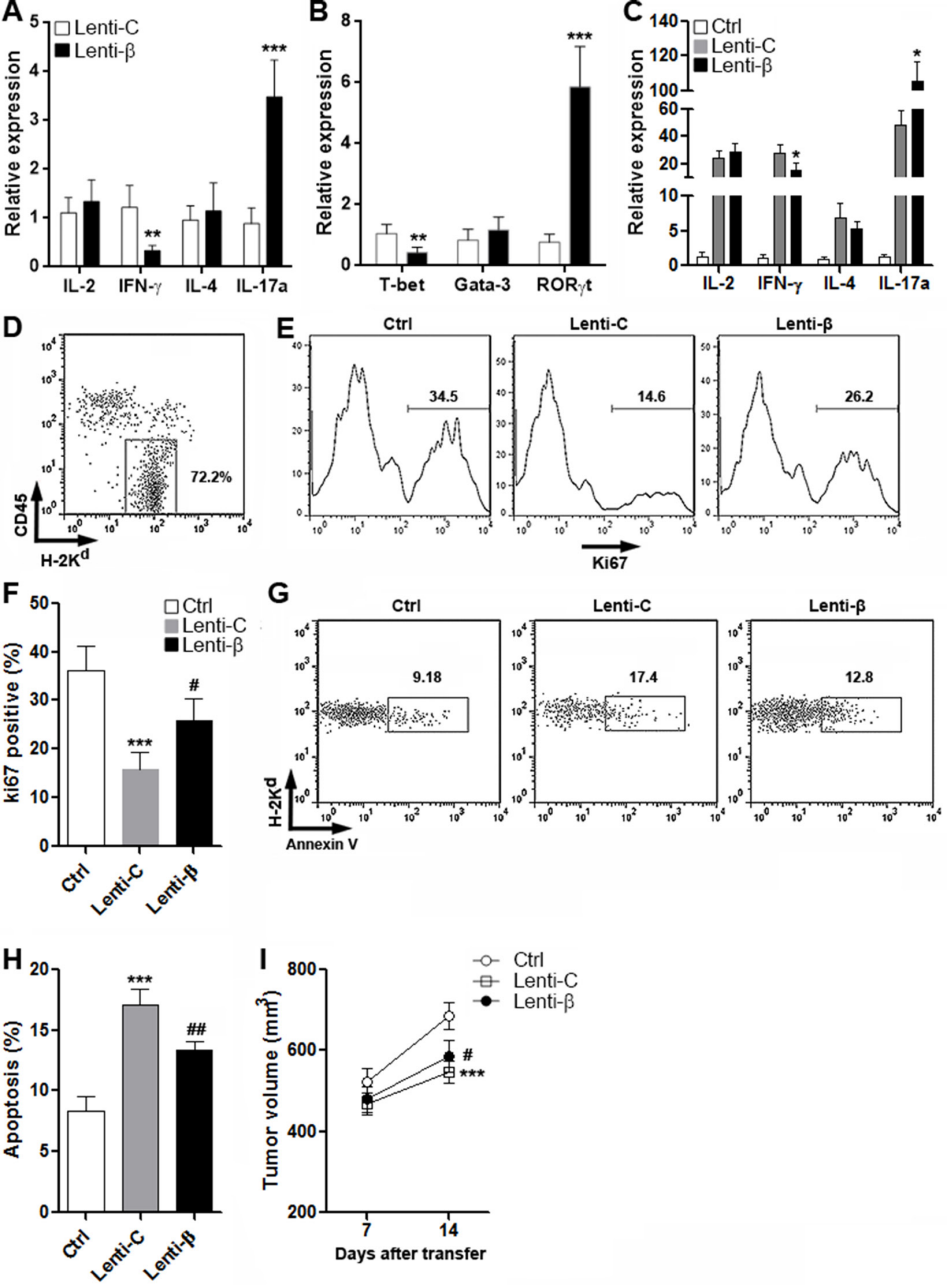
Enforced expression of β-catenin in CD4^+^ T cells favors growth of CRC grafts (**A** and **B**) Expression of cytokines (A) and master regulators (B) in donor-derived intratumoral CD4^+^ T cells were assessed by real-time PCR. Lenti-β: T cells transduced with β-catenin Lentiviral Activation Particles. Lenti-C: T cells transduced with control lentiviral particles. *N* = 6 per group. **p < 0.01; ***p < 0.001 compared with Lenti-C group. (**C**) Expression of indicated cytokines in CRC grafts. Ctrl: mice receiving PBS. Lenti-C: mice receiving T cells transduced with control lentiviral particles. Lenti-β: mice receiving T cells transduced with β-catenin Lentiviral Activation Particles. *N* = 6 per group. *p < 0.05 compared with Lenti-C group. (**D**) Gating strategy for CT26.CL25 cell detection in CRC grafts. (**E** and **F**) Proliferation of implanted CT26.CL25 cells. Histograms of Ki67 staining were shown in (E), and statistics was shown in (F). *N* = 6 per group. (**G** and **H**) Apoptosis of implanted CT26.CL25 cells was measured by flow cytometry. Representative dot plots of Annexin V staining were shown in (G), and statistics was shown in (H). N=6 per group. (**I**) Tumor volume after adoptive transfer. *N* = 10 per group. For F, H and I, ***p < 0.001 compared with Ctrl group. #*p* < 0.05, ##*p* < 0.01 compared with Lenti-C group.

To determine the CD4^+^ T cell reaction against CRC, CD4^+^ T cells were transduced with Lenti-C or Lenti-β and were then adoptively transferred into tumor-bearing Rag1^−/−^ mice. Cytokine production and master regulator expression in intratumoral T cells was measured two weeks after transfer. As shown in Figure 7A, in comparison with Lenti-C-transduced T cells, Lenti-β-transduced T cells showed lower IFN-γ and higher IL-17a production, while IL-2 and IL-4 were not altered. Consistently, reduced T-bet expression and increased RORγt were found in Lenti-β-transduced T cells (Figure 7B). These results were consistent with our *in vitro* data mentioned above. To evaluate the anti-CRC immune reaction in CRC grafts, cytokine production in the whole CRC graft tissue was determined two weeks after transfer. We found that the expression of IFN-γ was decreased while the expression of IL-17a was increased in the whole CRC graft tissue in mice receiving Lenti-β-transduced T cells (Figure 7C). To evaluate CRC graft growth, CD45^-^H-2K^d+^ CRC cells were detected for further characterization (Figure 7D). Ki67 staining of CRC cells indicated that Lenti-C-transduced T cells inhibited cancer cell growth, whereas Lenti-β-transduced T cells were weaker in doing so (Figure 7E and 7F and Supplementary Figure 3A). Apoptosis assay demonstrated that Lenti-β-transduced T cells were less capable of inducing cancer cell apoptosis than Lenti-C-transduced T cells (Figure 7G and 7H and Supplementary Figure 3B). Moreover, measurement of CRC graft size showed larger tumor volume in mice receiving Lenti-β-transduced T cells, compared with mice receiving Lenti-C-transduced T cells (Figure 7I). In general, our data indicated that excessive Wnt signaling weakened anti-cancer reaction of CD4^+^ T cells, and favored CRC graft growth.

## DISCUSSION

Wnts are crucial for cell proliferation, survival, migration and polarity, specification of cell fate, and self-renewal of stem cells [25]. The canonical Wnt pathway is a central mechanism in cancer biology, either beneficial or detrimental to the prognosis of cancer patients [26]. In CRC, activation of Wnt pathway has also been linked to carcinogenesis [27]. For example, expression of Wnt3, Wnt3a and Wnt10a are increased in CRC [18–20]. Wnt2 and Wnt5a expression are also elevated in colon cancer compared to normal colon and during the progression from adenoma to carcinoma [28, 29]. In contrast, the expression of other Wnts, including Wnt2b, 4, 7b, and 10b, does not change, as they remain strongly expressed in both normal colon and colon cancer [21]. Our data was consistent with above reports, showing higher Wnt3, Wnt3a, Wnt5a and Wnt10a in most cell lines tested. The CT26.CL25 cell-derived subcutaneous tumor grafts also expressed significant Wnt proteins.

Some immune cells expressing Wnt receptors could be target cells of Wnt proteins. Indeed, Wnt proteins induce changes of immune cell viability, differentiation and functions [30]. Due to the critical role of T cells in tumor immunity, Wnt signaling in T cells was focused in our research. Our study was inspired by a recent report demonstrating elevated β-catenin expression in CRC-infiltrating T cells [31]. We speculated that CRC cells could be the source of various Wnt ligands which activate canonical Wnt signaling in T cells. Based on a previous study [22], we found that expression of almost all FZDs in splenic naïve T cells were very weak except for FZD-6, and intratumoral T cells markedly increased FZD-3, FZD-5 and FZD-7. Hence, our data suggests higher FZD expression in CRC-reactive intratumoral T cells. However, future study will be necessary to test the antigen specificity of intratumoral T cells. Furthermore, other FZDs that have not been tested here, such as FZD-1, FZD-2 and FZD-8, should be evaluated in future.

We also found that intratumoral T cells had high levels of active β-catenin and total β-catenin, suggesting activation of Wnt signaling. However, due to insufficient information about engagement of Wnt ligands with specific FZD receptors, it is difficult for us to speculate which Wnt ligand or which FZD is predominant for suppressing T cell function. Probably different Wnt ligands simultaneously bind to different FZDs to synergistically induce Wnt signaling.

Our *in vitro* study suggests that CRC cells indeed stimulate canonical Wnt signaling in activated T cells. Interestingly, although TCR signaling was reported to stabilize β-catenin in T cells [32], our study did not show the same result. In our study, agonistic antibodies could not accumulate β-catenin, in accordance with a previous report indicating constitutive degradation of β-catenin in both resting and CD3/CD28 antibody-primed T cells [23]. Hence, it seems that activation of Wnt signaling unlikely resulted from TCR or CD3 signaling.

Studies indicate that Wnt/β-catenin signaling is a key regulator of T cell development at various stages of thymocyte differentiation [30, 33]. However, the exact significance of Wnt/β-catenin signaling for mature T cell immunity is still controversial. Inconsistent or even contradictory results have been generated in the past decades [24, 34–39], reflecting the subtle role of Wnt signaling in regulating T cell function. It is very likely that the effect of Wnt signaling is differential in distinct subpopulations of T cells. Regardless the previous inconsistent data, the inhibitory effect of Wnt signaling on T cell function has been repeatedly observed [23, 31, 39]. We also showed that CRC predominantly impair Th1 function. Interestingly, CRC-induced Wnt signaling increased IL-17a and RORγt expression in CD4^+^ T cells, which was consistent with two previous research [36, 40] but in contrast to to another two studies [41, 42]. It is possible that the comprehensive effect of CRC-derived Wnt ligands favors Th17 differentiation. It is also likely that CRC cells produce other cytokines and soluble mediators which work with Wnt ligands to induce Th17 polarization. Further research on the significance of Wnt signaling in Th17 polarization is necessary.

Our *in vivo* study using enforced β-catenin expression in CD4^+^ T cells further confirmed the *in vitro* data. The lower IFN-γ production in β-catenin-overexpressed T cells was accompanied by higher proliferation and less apoptosis of CRC cells, suggesting that Wnt signaling impaired CD4^+^ T cell-mediated anti-CRC immunity. In addition, IL-17a was reported to facilitate colon cancer development, promoting cancer-elicited inflammation and preventing cancer cells from immune surveillance [43–45]. Therefore, Wnt signaling not only inhibits Th1 cell-mediated CRC cell death, but also enhances Th17 cell-mediated CRC growth. We did not use CD8^+^ T cells for *in vivo* study because we did not see substantial functional changes of CD8^+^ T cells *in vitro*. However, we plan to test them in a similar system in the near future.

It should be noted that Wnt5a, a protein triggering non-canonical Wnt signaling, is also expressed in CRC cell lines, although it seems that its expression level is relatively weaker than other Wnt proteins. Non-canonical Wnt signaling has been shown to play roles in T cell development [46–48] but its role in mature T cell function is not clear. In addition, non-canonical Wnt signaling might play a critical role in CRC carcinogenesis. In particular, Wnt5a can induce an antagonistic signaling against canonical Wnt signaling [49] so it could inhibit CRC initiation or progression. However, due to its relatively low expression and the complexity of Wnt signaling, it is still hard to speculate the exact role of Wnt5a in CRC. More carefully designed experiments are needed in future.

Taken together, we are the first to indicate that CRC-derived Wnt ligands can influence T cell immunity in the tumor microenvironment. Our study shed new lights on the study of enhancing anti-CRC T cell immunity and might be beneficial for developing novel therapeutic interventions.

## MATERIALS AND METHODS

### CRC cell line culture

CRC cell line CT26.CL25 (mouse), HT-29, SW480 and HCT-116 cells (human) were obtained from the American type culture collection. Cells were cultured in Dulbecco-modified essential media (DMEM, Gibco) containing 4.5 g/l glucose supplemented with 10% fetal bovine serum (FBS, Hyclone), 3.44 mg/ml L-glutamine and 100 mg/ml of penicillin-streptomycin.

### Mouse CRC cell implantation model

All animal experiments were conducted in compliance with institutional guidelines and Jilin University Guidelines for the Use of Animals. All animal procedures were approved by Jilin University Animal Care and Use Committee. Eight-to-ten week old male BALB/C and Rag1^−/−^ mice (C57BL/6J background) were obtained from the Weitonglihua(Beijing) Laboratory Animal Technology Co., Ltd.. Rag1^−/−^ mice were subcutaneously (s.c.) inoculated with 0.5 × 10^6^ CT26.CL25 cells in 0.2 ml phosphate-buffered saline (PBS) at the lower left flank. Four weeks after inoculation, the mice were euthanized by inhalation of carbon dioxide for an average of 5 min. Tumor volume was measured according to the standard formula 1/2 × L × W^2^.

### Adoptive transfer of T cells

CD4^+^ and CD8^+^ T cells were sorted from BALB/C mouse axillary and inguinal lymph nodes using EasySep CD4^+^ T cell isolation Kit and CD8^+^ T cell isolation Kit (both from Miltenyi Biotec), respectively, according to the manufacturer's manual. In some experiments, the donor BALB/C mice were inoculated with 1 × 10^6^ CT26.CL25 cells at the left flank, and T cells were sorted two weeks after inoculation followed by *in vitro* expansion and lentiviral transduction as mentioned below. 1 × 10^7^ T cells (total T cells or CD4^+^ T cells) were retro-orbitally injected into tumor-bearing Rag1^−/−^ mice two weeks after inoculation of CT26.CL25 cells. The adoptive transfer was performed every 3 days for a week.

### Isolation of T cells and CT26.CL25 cells from implanted tumors

The tumor-bearing mice were anesthetized with isoflurane before perfusion with 10 ml ice-cold PBS from the heart. Fresh tumor tissues were cut into about 1 mm^3^ pieces and digested at 37°C for 30 min in 2 ml of RPMI 1640 supplemented with 0.05% collagenase Type IV (Sigma-Aldrich), 0.002% DNase I (Roche) and 20% FBS (Hyclone). Digested tumor tissues were then pressed through a 70-μm mesh to prepare single cell suspensions. For intratumoral T cell isolation or detection, the single cell suspension was carefully layered onto equal volume of Ficoll-Paque reagent (GE Healthcare) followed by centrifugation at 400 g for 30 min at room temperature. Mononuclear cells were then collected from the interface, washed in 3 volumes of PBS, and were incubated in 3 ml of Tris-NH_4_Cl solution for 3 min to lyse residual red blood cells. Cells were washed with PBS twice before sorting or detection T cells using flow cytometry. For CT26.CL25 cell detection, the single cell suspensions were directly stained with indicated antibodies or markers followed by flow cytometry analysis.

### Western blot

The cells, normal mouse colon tissue and normal mouse skin tissue were lysed in RIPA buffer containing protease inhibitor cocktail (Sigma-Aldrich). The following antibodies were used: anti-β-actin (ab8226), anti-Wnt2b (ab178418), anti-Wnt3 (ab32249), anti-Wnt3a (ab28472), anti-Wnt4 (ab91226), anti-Wnt5a (ab72583), anti-Wnt7b (ab94915), anti-Wnt10a (ab106522), anti-FZD-5 (ab75234), and anti-FZD-7 (ab64635) were purchased from Abcam. Anti-active β-catenin (D13A1) and anti-total β-catenin (D10A8) were purchased from Cell Signaling Technology. Membranes were developed with SuperSignal West Pico Chemiluminescent Substrate (Thermo Scientific) and the optical density was analyzed using a Biospetrum 500 imaging system.

### RNA extraction, cDNA synthesis and real-time PCR

Total RNA was extracted from cells or tissues using the RNeasy Mini Kit (Qiagen). One microgram of total RNA from each sample was transcribed into cDNA using SuperScript^®^ III First-Strand Synthesis System (Invitrogen) following the manufacturers’ instruction. qRT-PCR was performed using Fast SYBR^®^ Green Master Mix (Invitrogen) on a 7300 qRT-PCR System (Invitrogen). Data was analyzed with 7300 system software. Primer sequences for each gene are as follows: β-actin (5′-AGAGGGAAATCGTGCGTGAC-3′ and 5′-CAATAGTGATGACCTGGCCGT-3′). T-bet (5′-ACCGCTTATATGTCCACCCA-3′ and 5′-GAGAGACTGCAGGACGATCA-3′). Gata3 (5′-CCCATTACCACCTATCCGCC-3′ and 5′- GTTCACACACTCCCTGCCTT-3′). Foxp3(5′- TTGGCCAGCGCCATCTT-3′ and 5′-TGCCTCCTCCAGAGAGAAGTG-3′). RORc (5′-CACGGCCCTGGTTCTCAT-3′ and 5′-GCAGATGTTCCACTCTCCTCTTCT-3′). IL-2 (5′-CCTGAGCAGGATGGAGAATTACA-3′ and 5′-TCCAGAACATGCCGCAGAG-3′). IL-4 (5′-CTTTCGGGCTTTTCGATGCC-3′ and 5′-TCAGTGATGTGGACTTGGACTC-3′). IL-17a (5′-AGTCCAGGGAGAGCTTCATCT-3′ and 5′-CACGCTGAGCTTTGAGGGAT-3′). IFN-γ (5′-TGAACGCTACACACTGCATCTTGG-3′ and 5′-CGACTCCTTTTCCGCTTCCTGAG-3′). Perforin(5′-CTGGCAGGGACGATGACCT-3′ and 5′-GGGAACCAGACTTGG GAGC-3′). Granzyme B (5′-ATCAAGGATCAGCAGCCTGA-3′ and 5′-TG ATGTCATTGGAGAATGTCT-3′). TNF-α (5′-GCCTCTTCTCATTCCTGCT

TG-3′ and 5′-CTGATGAG AGGGAGGCCATT-3′). PCR conditions used for all primer sets were as follows: 95°C hot start for 10 min, followed by 40 amplification cycles of 95°C for 30 s (denaturing), 60°C for 1 min (annealing, extension and detection). Relative abundance of RNA was analyzed using 2^−ΔΔCt^ method.

### Flow cytometry analysis

The following anti-mouse antibodies or reagents were used for detection and sorting of T cells and CT26.CL25 cells: APC anti-CD45 (30-F11), FITC anti- H-2K^d^ (SF1-1.1), PE anti-TCRβ (H57-597), APC-Cy7 anti-CD4 (GK1.5), PE-Cy7 anti-CD8a (53-6.7), FITC anti-IFN-γ (MP6-XT22) and Alexa Fluor^®^ 488 anti-IL-17a (TC11-18H10) were purchased from BD Pharmingen. Anti-FZD-3, anti-FZD-4 and anti-FZD-6 were purchased from R&D Systems. Anti-active β-catenin (D13A1) was purchased from Cell Signaling Technology. PE anti-Ki67 was purchased from Biolegend. PE Annexin V and carboxyfluorescein diacetate succinimidyl ester (CFSE) were purchased from BD Pharmingen.

For cell surface staining, cells were incubated with corresponding antibodies in PBS for 30 min on ice before analysis on a BD LSRII flow cytometer. Dead cells that were stained by propidium iodide (PI, 5 μg/ml) were excluded. For intracellular cytokine staining, cells were fixed and permeabilized with BD Cytofix/perm and Perm/wash buffer according to the manufacturer's protocol, respectively. Then cells were stained at room temperature for 30 min with FITC anti-IFN-γ or Alexa Fluor^®^ 488 anti-IL-17a. For active β-catenin staining, cells were fixed for 10 min at 37°C with BD Cytofix/perm buffer followed by permeabilization with 90% methanol for 30 min on ice. Then cells were stained for 30 min at room temperature with anti-active β-catenin antibody (1:50). Cells were washed with PBS once before incubation with FITC goat anti-rabbit IgG (BD Pharmigen) for 0 min at room temperature. For cell apoptosis assay, cells were stained with 2 μg/ml PI and PE Annexin V (BD Pharmigen) according to the manufacturer's instruction. All samples were detected on a BD LSRII flow cytometer (BD Bioscience). All flow cytometry data was analyzed with Flowjo 7.6.1 software. Cell sorting was performed on a BD FACSAria cell sorter based on cell surface marker staining.

### Co-culture of T cells with CT26.CL25 cells

T cells were treated in the presence or absence of agonistic antibodies before co-culture. Briefly, a 96-well plate was pre-coated with 5 μg/ml anti-CD3 monoclonal antibody (17A2, eBiscience). 1 × 10^5^ sorted CD4^+^ or CD8^+^ T cells were seeded into each pre-coated well and incubated for two days in the presence of 2 μg/ml anti-CD28 antibody (37.51, eBioscience). Then these T cells were transferred into a 24-well plate pre-coated with 5 μg/ml anti-CD3 monoclonal antibody. Meanwhile, 2.5 × 10^5^ CT26.CL25 cells were seeded into each Transwell insert (0.4 μm pore. Corning Costar), and the inserts were placed in the T cell-containing 24-well plate. After 48 h co-culture, T cells were collected and subject to further processing. In some experiments, recombinant mouse Dickkopf 1 (Dkk1, R&D Systems), an inhibitor of Wnt signalling, was added at 400 ng/ml in the co-culture.

For T cell proliferation assay, T cells were labelled with 2 μM CFSE before stimulation with agonistic antibodies and co-culture as mentioned above. CFSE dilution in T cells were then evaluated by flow cytometry.

For intracellular cytokine staining, the cancer cell-containing Transwell inserts were removed after co-culture. T cells were re-stimulated with 20 ng/ml phorbol myristate acetate (PMA) and 1 μM Ionomycin in the presence of 5 μg/ml brefedin A and 5 μg/ml monensin for 4 h before flow cytometry analysis.

### Lentiviral transduction

β-catenin Lentiviral Activation Particles were purchased from Santa Cruz Biotechnology. 1.5 × 10^6^ CD4^+^ T cells were expanded *in vitro* for 7 days in 6-well plates with plate-bound anti-CD3 antibody and soluble anti-CD28 antibody in the presence of 100 U/ml recombinant mouse IL-2 (R&D systems). At day 5 after expansion, polybrene was added into the medium to 5 μg/ml. Then T cells were transduced with lentiviral particles at an MOI of 20 overnight. Cells were washed and expanded for additional 2 days. Then T cells were washed with PBS and were subject to further experiments.

### Statistics

Most data was presented as mean ± SEM and analyzed by statistical software (Prism 6.0, GraphPad Software). Student's *t* test or one-way ANOVA were used for comparison of mean between the groups. *P* values < 0.05 were considered significant.

## SUPPLEMENTARY MATERIALS FIGURES


