# Supplementary material for: Colorectal cancer cells suppress CD4+ T cells immunity through canonical Wnt signaling
Source: Oncotarget. 2017 Jan 27;8(9):15168–81. doi: 10.18632/oncotarget.14834 (PMC5362476; doi:10.18632/oncotarget.14834)
Supplement: Supplementary file 1 [file oncotarget-08-15168-s001.pdf]

Colorectal cancer cells suppress CD4$^+$ T cells immunity through canonical Wnt signaling

Supplementary Materials

SUPPLEMENTARY INFORMATION

MATERIALS AND METHODS

Flow cytometry and cell sorting

Digestion of implanted tumors were conducted the same way as in the manuscript. The following antibodies were used for detection and isolating corresponding cell types: FITC anti-H-2Kb (AF6-88.5.5.3), APC anti-CD31 (390), PE anti-CD140a (APA5), APC-eFluor® 780 anti-F4/80 (BM8) and PE-Cy7 anti-CD11c (N418) were purchased from eBioscience. Cells were sorted on a BD FACSAria cell sorter based on cell surface marker staining.

Real-time PCR

The following primers (Forward and Reverse) were used for relative quantification of mRNA levels: Wnt3a (5′TGGCTCCTCTCGGATACCTC3′and 5′GGGATGCTGGCACAGAGAAT3′); Wnt3 (5′CACAACACGAGGACGGAGAA3′ and 5′GGATCCAGCCGCACAATCTA3′); Wnt5a (5′GGCTTTGGCCACGTTTTTCT3′ and 5′GAGAGGCTGTGCACCTATGA3′); Wnt10a (5′ACAAAGTCCCCTACGAGAGC3′ and 5′GCAAGCCTTCAGTTTACCCAG3′).

Immunohistochemistry and terminal deoxynucleotidyl transferase dUTP nick end labeling (TUNEL)

The mice were transcardially perfused with 4% paraformaldehyde to fix tissue and to remove blood cells in blood vessels. Then CRC tumors were isolated and embedded in paraffin using a Leica APS300. Five-micron cross sections were prepared and antigen retrieval was performed by boiling sections in sodium citrate buffer (10 mM sodium citrate, 0.05% Tween 20, pH 6.0) for 30 min. Anti-CD3 antibody (Abcam, ab5690) or Anti-Ki67 antibody (Abcam, ab15580) was used for staining according to the manufacturer’s instructions. Horseradish Peroxidase-conjugated goat anti-rabbit IgG H&L (Abcam, ab6721) was added after three PBS washes. VECTASTAIN Elite ABC HRP Kit (Vector Laboratories) was used to indicate positive cells according to the manufacturer’s instruction, followed by Hematoxylin counterstain and glycerol mounting.

For TUNEL assay, tumor sections were labeled with DeadEnd™ Fluorometric TUNEL System (Promega) following the manufacturer’s manual. Sections were mounted with VECTASHIELD Antifade Mounting Medium with DAPI (Vector Laboratories). The sections were observed on a Leica DMIRE2 inverted fluorescent microscope.

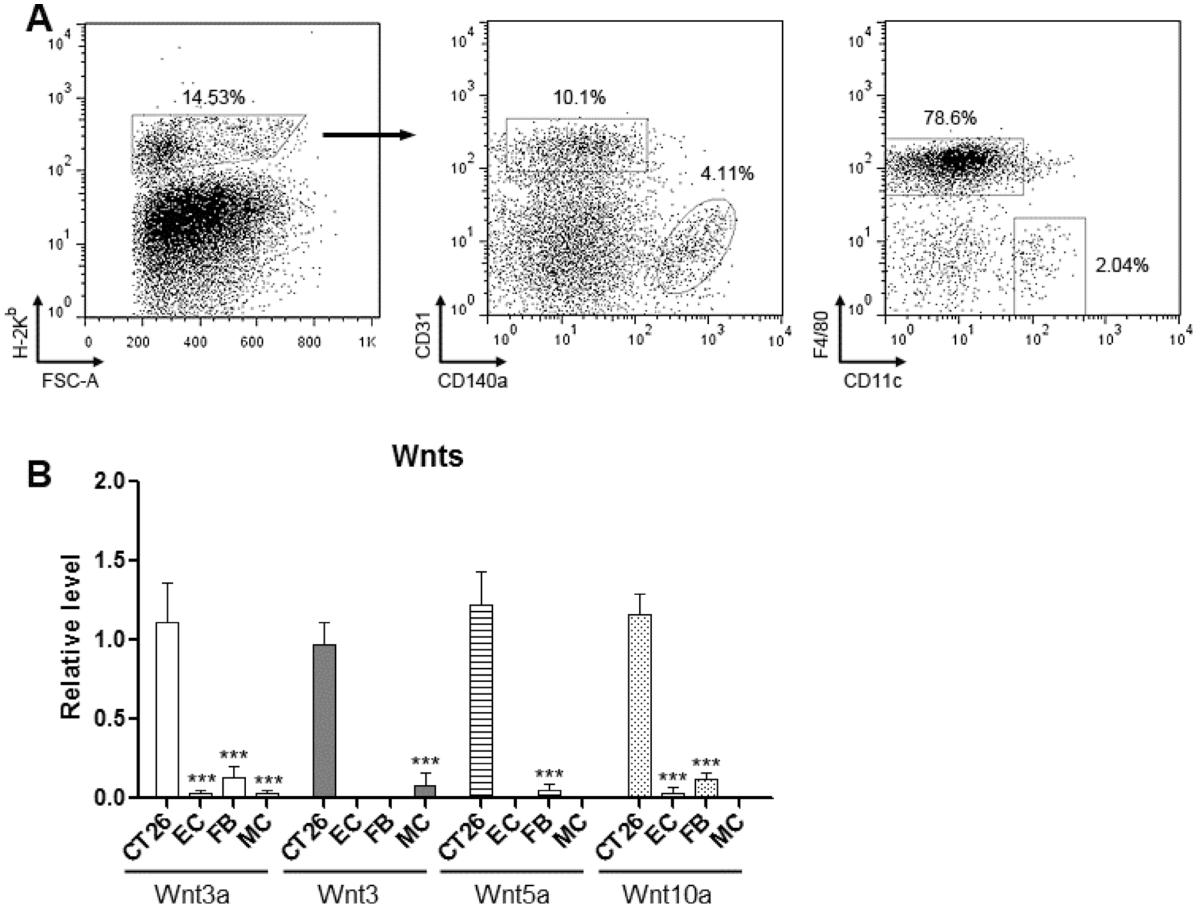

Supplementary Figure 1: Different non-colorectal cancer cell types express much less Wnts than colorectal cancer cells in the implanted colorectal cancer tumor. (**A**) Gating strategy for non-colorectal cancer cell types in the implanted colorectal cancer tumor. Digestion of implanted tumors were conducted as described in the manuscript. H-2k^b-postive host-derived cells were further gated for sorting of CD31-positive endothelial cells, CD140a-positive fibroblasts, F4/80-positive macrophages and CD11c-positive dendritic cells. H-2k^d-positive CT26.CL25 cells were sorted as described in the manuscript. (**B**) mRNA levels of Wnt3a, Wnt3, Wnt5a and Wnt10a in sorted host cell types were determined by real-time PCR and were compared with implanted CT26.CL25 cells. EC: endothelial cells. FB: fibroblasts. MC: macrophages. Expression of these Wnts was not detectable in dendritic cells so dendritic cell group was not shown. $N = 3$ per group. ***$p < 0.001$ compared with CT26.CL25 cells.

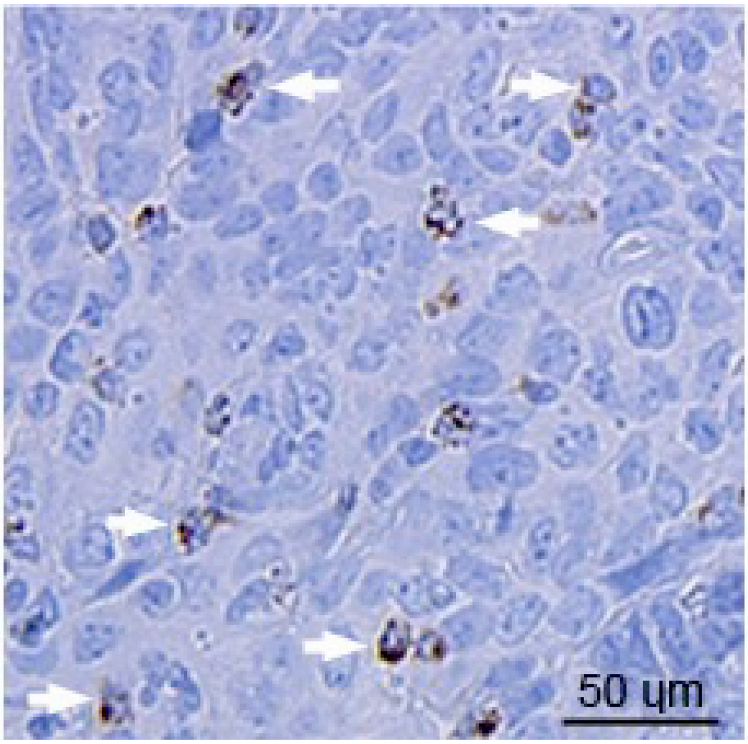

Supplementary Figure 2: T cell infiltrates in the implanted CRC tumor. White arrows indicate CD3-positive T cells in one section of implanted CRC tumors. This is a representative image of two independent tumor sections.

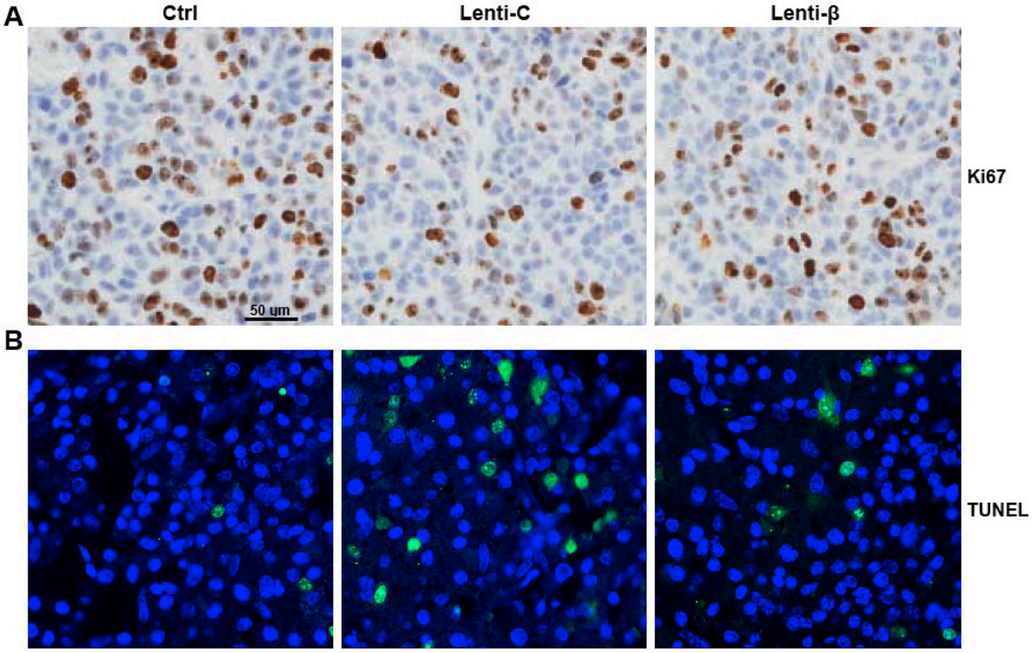

Supplementary Figure 3: Ki67 staining and TUNEL in implanted CRC tumors. (**A**) Immunohistochemistry for Ki67 in tumors. (**B**) TUNEL for tumors sections. Ctrl: mice receiving PBS. Lenti-C: mice receiving T cells transduced with control lentiviral particles. Lenti-β: mice receiving T cells transduced with β-catenin Lentiviral Activation Particles.
